# Identification of two new genetic loci for high-resolution genotyping of *Enterocytozoon bieneusi*

**DOI:** 10.1051/parasite/2025002

**Published:** 2025-01-31

**Authors:** Xinan Meng, Yonglin Ou, Wen Jiang, Yaqiong Guo, Lihua Xiao, Yaoyu Feng, Na Li

**Affiliations:** 1 State Key Laboratory for Diagnosis and Treatment of Severe Zoonotic Infectious Diseases, Key Laboratory for Zoonosis Research of the Ministry of Education, South China Agricultural University Guangzhou 510642 China; 2 Guangdong Laboratory for Lingnan Modern Agriculture, Center for Emerging and Zoonotic Diseases, College of Veterinary Medicine, South China Agricultural University Guangzhou 510642 China

**Keywords:** *Enterocytozoon bieneusi*, Multilocus sequence typing, Genetic marker, Genetic diversity

## Abstract

In addition to the ribosomal internal transcribed spacer (ITS) locus, four loci (MS1, MS3, MS4, and MS7) have been identified to develop multilocus sequence typing tools for high-resolution genotyping of *Enterocytozoon bieneusi* in previous studies. However, the use of only five loci was insufficient for population genetic analysis of *E. bieneusi* from diverse hosts. In this study, comparison of a clinical genome sequence (C44566) with the whole genome sequence of an *E. bieneusi* isolate (H348) in GenBank led to the selection of the hypothetical protein 1 (*hp1*) and tubulin 1 (*tub1*) loci. Further analysis of the two loci with 156 *E. bieneusi*-positive samples showed high sequence polymorphisms in ITS Groups 1–6 and 10. Altogether, 30 and 23 sequence types were identified at *hp1* and *tub1*, respectively. Genotyping based on the two loci confirmed the lack of genetic differentiation between Group 1 and Group 2 genotypes, as previously reported. Moreover, the genotypes in Groups 4 and 5 are more divergent from other genotypes within Groups 1–10. However, isolates in Group 11 and 12 could not be amplified at the *hp1* and *tub1* loci, supporting the previous conclusion of genetic uniqueness of the two genotype groups. The identified genetic markers and generated data could be used to develop a multilocus sequence typing tool for high-resolution genotyping of *E. bieneusi*, which would also have implications for understanding the taxonomy of *Enterocytozoon* spp., the public health significance of *E. bieneusi* in animals, and sources of *E. bieneusi* infections in humans.

## Abstract (fr)

En plus du locus ITS (espaceur interne transcrit) ribosomal, quatre loci (MS1, MS3, MS4 et MS7) ont été identifiés pour développer des outils de typage de séquence multilocus pour le génotypage haute résolution d’*Enterocytozoon bieneusi* dans des études précédentes. Cependant, l’utilisation de seulement cinq loci était insuffisante pour l’analyse génétique de la population d’*E. bieneusi* à partir de divers hôtes. Dans cette étude, la comparaison d’une séquence du génome clinique (C44566) avec la séquence du génome entier d’un isolat d’*E. bieneusi* (H348) dans GenBank a conduit à la sélection des loci de la protéine hypothétiques 1 (*hp1*) et de la tubuline 1 (*tub1*). Une analyse plus poussée des deux loci avec 156 échantillons positifs à *E. bieneusi* a montré des polymorphismes de séquence élevés dans les groupes ITS 1 à 6 et 10. Au total, 30 et 23 types de séquence ont été identifiés respectivement à *hp1* et *tub1*. Le génotypage basé sur les deux loci a confirmé l’absence de différenciation génétique entre les génotypes du groupe 1 et du groupe 2 comme trouvé précédemment. De plus, les génotypes des groupes 4 et 5 sont plus divergents des autres génotypes des groupes 1 à 10. Cependant, les isolats des groupes 11 et 12 n’ont pas pu être amplifiés aux loci *hp1* et *tub1*, ce qui étaye la conclusion précédente de l’unicité génétique de ces deux groupes de génotypes. Les marqueurs génétiques identifiés et les données générées pourraient être utilisés pour développer un outil de typage de séquences multilocus pour le génotypage haute résolution d’*E. bieneusi*, ce qui devrait également avoir des implications pour la compréhension de la taxonomie d’*Enterocytozoon* spp., l’importance d’*E. bieneusi* pour la santé publique chez les animaux et les sources d’infections par *E. bieneusi* chez l’homme.

## Introduction

*Enterocytozoon bieneusi* is emerging as an important zoonotic pathogen [19, 26]. It infects humans and various animals and is responsible for over 90% of reported cases of human microsporidiosis [8]. *Enterocytozoon bieneusi* infections are mostly limited to the gastrointestinal tract; in those with immunocompromising conditions, it usually causes severe or chronic diarrhea, malabsorption, and wasting [7].

Genotyping based on the internal transcribed spacer (ITS) region of the rRNA gene is the most common method for *E. bieneusi* typing. Fifteen phylogenetic groups including over 500 *E. bieneusi* genotypes have been identified based on sequence analysis of the ITS locus [10, 23]. ITS genotypes in Groups 1 and 2 have been found in a broad range of hosts and are probably responsible for most zoonotic or cross-species *E. bieneusi* infections, while ITS genotypes in Groups 3–15 have stronger host specificity [15]. Among them, the Group 11 genotypes are genetically divergent from other genotype groups of *E. bieneusi*, probably representing a different *Enterocytozoon* sp. This has been supported by sequence analysis of the 16S rRNA gene and two other more conservative markers *ck1* and *swp1*, which have recently been developed for assessment of genetic diversity within the *E. bieneusi* species complex [20].

The multilocus sequence typing (MLST) technique is a high-resolution typing method of *E. bieneusi* from humans and various animals. This technology was established because single locus-based typing method using ITS have insufficient resolution to discriminate *E. bieneusi* isolates with complex hereditary characteristics, and to assess the elusive reproduction or transmission modes of this pathogen. Therefore, additional genetic markers should be characterized to identify subtle genetic variability among *E. bieneusi* isolates from different hosts and environments [5]. Four mini- and microsatellites (MS1, MS3, MS4, and MS7) were initially tested and determined to be appropriate for high-resolution typing of *E. bieneusi* [5]. However, the high sequence polymorphisms observed at these specific loci posed a challenge in designing conservative primers, leading to low amplification efficiency for genotypes belonging to Groups 2–11 [5, 17]. In addition, the use of only five loci was insufficient for population genetic analysis of *E. bieneusi* genotypes.

In this study, we aim to identify more genetic markers as additional loci for high-resolution typing of *E. bieneusi* isolates, especially genotypes in Groups 2–11. The potential for further research on population genetics of *E. bieneusi* in humans and animals is enhanced by the discovery of these new genetic markers. These markers offer valuable opportunities to investigate the transmission dynamics, host specificity, and potential zoonotic transmission of *E. bieneusi*, thereby advancing our understanding of its epidemiology and facilitating the development of effective control strategies.

## Materials and methods

### Ethics

The research protocol was approved by the Research Ethics Committee of South China Agricultural University. The samples used for the study were handled in compliance with the regulations of the Chinese Laboratory Animal Administration Act of 2017.

### Samples

A total of 156 *E. bieneusi* samples were used in this study. These samples were obtained from previous epidemiological studies of *E. bieneusi* infections in humans and animals conducted in China, Kenya, Nigeria, Peru, Portugal, and the USA. The samples were selected to be representative of diverse host species and geographic regions to ensure a comprehensive analysis of genetic diversity. The samples were collected from various hosts, including humans, cattle, bamboo rats, dogs, civets, and horses [1, 3, 4, 6, 12, 16, 21, 28, 31, 32]. DNA extraction was performed on these samples using a FastDNA Spin Kit for Soil (MP Biomedical, Irvine, CA, USA), following the protocol described [9]. The extracted DNA was stored at −80 °C until further analysis. All 156 samples were tested positive for ITS PCR, with 36 identified genotypes belonging to Groups 1–6 and Groups 10–12 (Table S1) [10].

### Selection of new polymorphic loci

To identify polymorphic loci, we utilized the genome sequence data of the ITS genotype WL4 (isolate C44566 from a human clinical sample) and the whole genome sequence of *E. bieneusi* (H348) (NCBI: txid481877) [2] obtained from GenBank. These sequences were aligned using MAUVE 2.4.0 (http://darlinglab.org/mauve/mauve.html) to identify conserved regions, while also pinpointing loci with potential sequence variability. Two loci of hypothetical protein 1 (*hp1*, EBI_21704) and tubulin 1 (*tub1*, EBI_21729) were selected based on their polymorphic nature and suitability for sequence-based discrimination of genotypes. Nested PCR primers were designed based on the conserved flanking regions of the two loci, ensuring high amplification success across various genotypes. The loci were initially validated by performing nested PCR and sequence analysis on a set of *E. bieneusi*-positive samples to confirm their utility for genotyping.

### PCR analysis

A nested PCR assay was designed to amplify the two new genetic loci. Table 1 displays the primer sequences for the two loci in nested PCR. For the PCR reaction, the mixture included 1 μL of DNA for the primary PCR or 2 μL of primary PCR product for the secondary PCR, 25 μL of DreamTaq PCR Master Mix (2 ×) (Thermo Fisher Scientific, Waltham, MA, USA), 100 nM forward primers, and 100 nM reverse primers in a 50 μL reaction. The nested PCR amplification was conducted with the following cycling condition: 94 °C for 5 min, 35 cycles of 94 °C for 45 s, 55 °C for 45 s, and 72 °C for 60 s, and 72 °C for 7 min. The procedure for the secondary PCR was identical to the primary PCR. Each sample was analyzed twice by PCR at each genetic locus. The secondary PCR products were examined using agarose gel electrophoresis and ethidium bromide staining.

**Table 1:** PCR primers used in the genetic characterization of the hypothetical protein 1 (*hp1*) and tubulin 1 (*tub1*) loci in *Enterocytozoon bieneusi.*

Locus	Gene tag	Description	Primers (5′ 3′)	Annealing temp (°C)	Expected product size (bp)
*hp1*	EBI_21704	Hypothetical protein 1	F1, TTGTGGATTTCCTGTCAAAGA	55	775
			R1, TAAGGATGTAGTTACAAATGGAT		
			F2, ATGATGTTATTTGCGATGATGT	55	675
			R2, GTTCTAATTTTAATGGAACAAATGGA		
*tub1*	EBI_21729	Tubulin 1	F1, CTGCAACGCGTTGTTCTT	55	811
			R1, CGTAATTTAACATATGCAACCTCA		
			F2, AGTATTCACTCAGAAGCGATT	55	746
			R2, TTAGTGGTTTAACTTTAGCAACATT		

### Sequence analysis

All positive secondary PCR products were subjected to bi-directional sequencing using an ABI 3730 Genetic Analyzer (Applied BioSystems, Foster City, CA, USA) at BioSune Biotechnology (Shanghai, China). The obtained sequences were assembled using ChromasPro 2.1.6 (http://technelysium.com.au/ChromasPro), edited by BioEdit 7.1.3 (http://www.mbio.ncsu.edu/BioEdit/bioedit), and aligned with reference sequences from GenBank using ClustalX 1.81 (http://www.clustal.org/). A maximum likelihood (ML) tree was constructed by MEGA7 (https://www.megasoftware.net/) with the general time-reversible model. The reliability of cluster formation was evaluated using the bootstrap method with 1000 replicates.

## Results

### Polymorphic loci selected

The two polymorphic loci *hp1* and *tub1* were selected by comparing the whole genome sequences between an *E. bieneusi* isolate C44566 and an *E. bieneusi* isolate H348 [2]. In the 675-bp *hp1* sequence, there was 98% (660/675) sequence identity between the two isolates within the nucleotide range of 72765–73439 (ABGB01000015). Similarly, in the 746-bp *tub1* sequence, there was 99% (741/746) sequence identity between the two isolates within the nucleotide range of 73796–74541 (ABGB01000015).

### PCR amplification efficiency of the two newly identified loci

**Figure 1:**
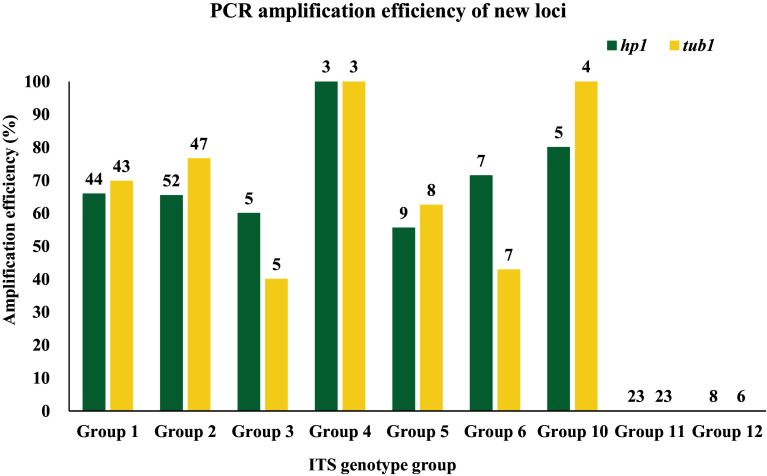
Efficiency of nested PCR analysis of the *hp1* and *tub1* loci from *Enterocytozoon bieneusi*. The amplification efficiency of Group 1 (65.9%), Group 2 (65.4%), Group 3 (60.0%), Group 4 (100.0%), Group 5 (55.6%), Group 6 (71.4%), and Group 10 (80.0%) at the *hp1* locus were all greater than 50%. The amplification efficiency of Group 1 (69.8%), Group 2 (76.6%), Group 4 (100.0%), Group 5 (62.5%), and Group 10 (100.0%) at the *tub1* locus were all greater than 50%. The number shown at the top of the bar chart indicates the size of samples analyzed.

At the *hp1* locus, the amplification efficiency was determined to be 53.2% (83/156). Isolates from Group 1 (65.9%, 29/44) and Group 2 (65.4%, 34/52) exhibited similar amplification efficiencies at this locus, as shown in Table S2. In contrast, both Group 11 and Group 12 genotypes failed to generate the desired target products (Fig. 1).

At the *tub1* locus, the amplification efficiency was 56.8% (83/146). The isolates from Group 1 (69.8%, 30/43) and Group 2 (76.6%, 36/47) demonstrated comparable PCR amplification efficiency, as shown in Table S2. Similar to the *hp1* locus, genotypes in Groups 11 and 12 were not amplified at the *tub1* locus (Fig. 1).

### Sequence polymorphism and genetic relationship of the *hp1* locus

**Figure 2:**
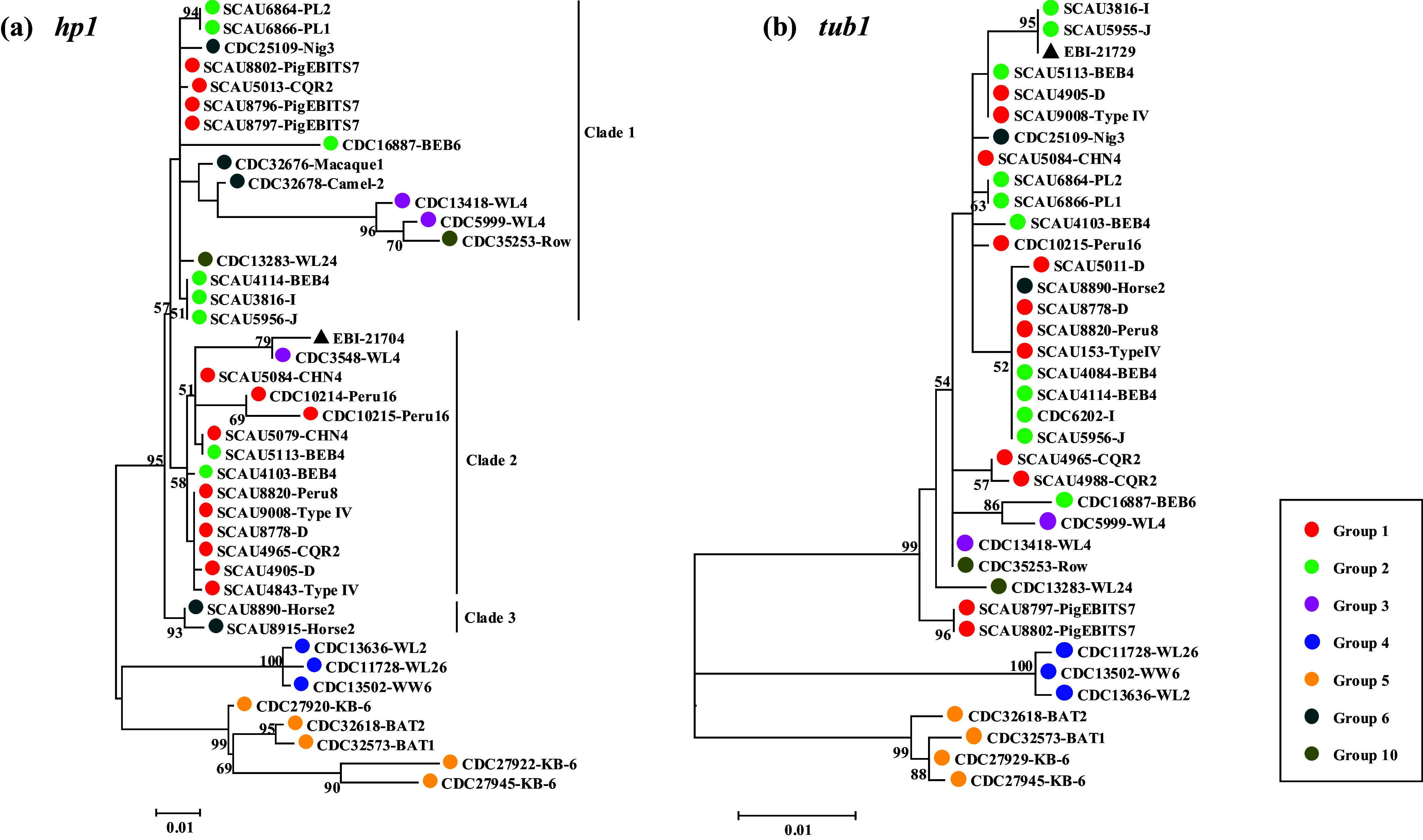
Maximum likelihood trees constructed using representative sequences of the *hp1* (a) and *tub1* (b) loci from *Enterocytozoon bieneusi.* Bootstrap values greater than 50% from 1000 replicates are shown on nodes. Circles of different colors (red, light green, magenta, blue, yellow, dark cyan, and olive) represent isolates from ITS Groups 1–6 and 10, respectively. The black triangle represents the reference sequence from GenBank.

A total of 83 *E. bieneusi*-positive isolates were successfully amplified and sequenced at the *hp1* locus, resulting in the identification of 30 sequence types, as shown in Table S1. Within ITS Groups 1 and 2, the generated sequences exhibited variation from the reference sequence EBI_21704 by 1–27 single nucleotide polymorphisms (SNPs), without clear differentiation between the two groups (Table 2 and Fig. 2a). Among them, four genotypes in Group 1 (CQR2, CHN4, D, and Type IV) and one genotype in Group 2 (BEB4) produced different *hp1* sequences. Although there were only three *hp1* positive samples in Group 3, sequences of 1–28 SNPs and three sequence types were produced compared to the reference sequence (EBI_21704). Similarly, three ITS genotypes in Group 4 (WW6, WL2, and WL26) produced sequences of 34–38 SNPs and three sequence types compared to the reference sequence (EBI_21704). The three ITS genotypes (BAT1, BAT2, and KB-6) in Group 5 produced sequences with 28–46 SNPs and four sequence types compared to the reference sequence (EBI_21704). The four ITS genotypes in Group 6 (Nig3, Camel-2, Macaque1, and Horse2) produced sequences of 9–13 SNPs and three sequence types in five samples compared to the reference sequence (EBI_21704). In contrast, compared with the reference sequence, two ITS genotypes (WL24 and Row) in four samples in Group 10 produced sequences with 9 SNPs and 25 SNPs, respectively.

**Table 2:** Sequence types of *Enterocytozoon bieneusi* at the hypothetical protein 1 (*hp1*) and tubulin 1 (*tub1*) loci by internal transcribed spacer (ITS) genotype.

Group	ITS genotype (No. of samples)	No. of SNPs (No. of samples)	
		*hp1*	*tub1*
1	CHN4 (2)	2 SNPs (1)	4 SNPs (2)
		3 SNPs (1)	
	CQR2 (8)	3 SNPs (1)	5 SNPs (6)
		8 SNPs (5)	6 SNPs (1)
	D (9)	3 SNPs (6)	3 SNPs (4)
		4 SNPs (1)	4 SNPs (3)
			5 SNPs (2)
	Peru8 (1)	3 SNPs (1)	4 SNPs (1)
	Peru16 (3)	4 SNPs (3)	5 SNPs (2)
	PigEBITS7 (4)	7 SNPs (4)	9 SNPs (3)
	Type IV (6)	3 SNPs (5)	3 SNPs (2)
		4 SNPs (1)	4 SNPs (4)
2	BEB4 (8)	2 SNPs (5)	2 SNPs (5)
		5 SNPs (1)	3 SNPs (3)
		8 SNPs (2)	
	BEB6 (1)	27 SNPs (1)	11 SNPs (1)
	I (8)	8 SNPs (8)	0 SNP (5)
			4 SNPs (1)
	J (8)	8 SNPs (8)	0 SNP (7)
			4 SNPs (1)
	PL1 (8)	10 SNPs (6)	5 SNPs (7)
	PL2 (6)	10 SNPs (3)	5 SNPs (6)
3	WL4 (3)	1 SNP (1)*	5 SNPs (1)
		15 SNPs (1)	
		28 SNPs (1)	8 SNPs (1)
4	WL2 (1)	35 SNPs (1)	41 SNPs (1)
	WL26 (1)	38 SNPs (1)	41 SNPs (1)
	WW6 (1)	34 SNPs (1)	40 SNPs (1)
5	BAT1 (1)	34 SNPs (1)	36 SNPs (1)
	BAT2 (1)	34 SNPs (1)	35 SNPs (1)
	KB-6 (5)	28 SNPs (1)	34 SNPs (1)
		38 SNPs (1)	35 SNPs (2)
		46 SNPs (1)	
6	Camel-2 (1)	9 SNPs (1)	
	Horse2 (2)	10 SNPs (1)	4 SNPs (2)
		13 SNPs (1)	
	Macaque1 (1)	9 SNPs (1)	
	Nig3 (1)	10 SNPs (1)	5 SNPs (1)
10	Row (3)	25 SNPs (3)	5 SNPs (3)
	WL24 (1)	9 SNPs (1)	9 SNPs (1)

The reference sequences for the *hp1* and *tub1* loci in MicrosporidiaDB are EBI_21704 and EBI_21729, respectively.

* The sequence is likely from a Group 1 genotype due to co-infection of two genotypes.

Phylogenetically, the obtained sequences from the *hp1* locus showed partial clustering according to the ITS genotype groups (Fig. 2a). The largest cluster consisted of seven genotypes in Group 1 (CHN4, CQR2, D, PigEBITS7, Peru8, Peru16, and Type IV), six genotypes in Group 2 (I, J, BEB6, BEB4, PL1, and PL2), two genotypes in Group 3 (WL4 and WL5), four genotypes in Group 6 (Camel-2, Horse2, Macaque1, and Nig3), and two genotypes in Group 10 (WL24 and Row) (Fig. 2a). Among them, two genotypes in Group 1 (CQR2 and PigEBITS7), six genotypes in Group 2 (I, J, BEB6, BEB4, PL1, and PL2), two genotypes in Group 3 (WL4 and WL5), three genotypes in Group 6 (Camel-2, Macaque1, and Nig3), the genotypes WL24 and Row in Group 10 formed clade 1. Six genotypes in Group 1 (CHN4, CQR2, D, Peru8, Peru16, and Type IV), BEB4 in Group 2, WL4 in Group 3 formed clade 2. The genotype Horse2 in Group 6 formed a separate clade designated as clade 3. In addition, three genotypes (WW6, WL2, and WL26) in Group 4 and three genotypes in Group 5 (BAT1, BAT2, and KB-6) formed a sister cluster with the largest cluster (Fig. 2a).

### Sequence polymorphism and genetic relationship of the *tub1* locus

A total of 83 isolates were successfully amplified and sequenced at the *tub1* locus, resulting in the identification of 23 sequence types, as shown in Table S1. Within ITS Groups 1 and 2, the generated sequences exhibited variations from the reference sequence EBI_21729 by 0–11 SNPs, without a consistent differentiation between the two groups (Table 2 and Fig. 2b). Among them, three genotypes in Group 1 (CQR2, D, and Type IV) and three genotypes in Group 2 (BEB4, I, and J) each produced different *tub1* sequences. Although Group 3 had only two *tub1* positive samples, it produced sequences with 5 and 8 SNPs, and produced two *tub1* sequence types. Three ITS genotypes (WW6, WL2, and WL26) within Group 4 produced sequences with 40–41 SNPs and produced two *tub1* sequence types. Similarly, three ITS genotypes within Group 5 (BAT1, BAT2, and KB-6) produced sequences with 34–36 SNPs and produced four *tub1* sequence types, two ITS genotypes within Group 6 (horse2 and Nig3) produced sequences with 4 and 5 SNPs, respectively, and two ITS genotypes (WL24 and Row) in four samples in Group 10 produced sequences with 9 SNPs and 5 SNPs, respectively.

Phylogenetically, sequences generated at the *tub1* locus showed a clustering pattern not aligned with the ITS genotype groups (Fig. 2b). Among these sequences, the largest cluster consisted of seven genotypes from Group 1 (CHN4, CQR2, D, PigEBITS7, Peru8, Peru16, and Type IV), six genotypes from Group 2 (I, J, BEB6, BEB4, PL1, and PL2), two genotypes from Group 3 (WL4 and WL5), two genotypes from Group 6 (Horse2 and Nig3), and two genotypes from Group 10 (WL24 and Row) (Fig. 2b). Additionally, three genotypes from Group 4 (WW6, WL2, and WL26) and three genotypes from Group 5 (BAT1, BAT2, and KB-6) formed distinct clusters that were separate from the largest cluster (Fig. 2b).

### Genetic relationship based on concatenated sequences of three loci

**Figure 3:**
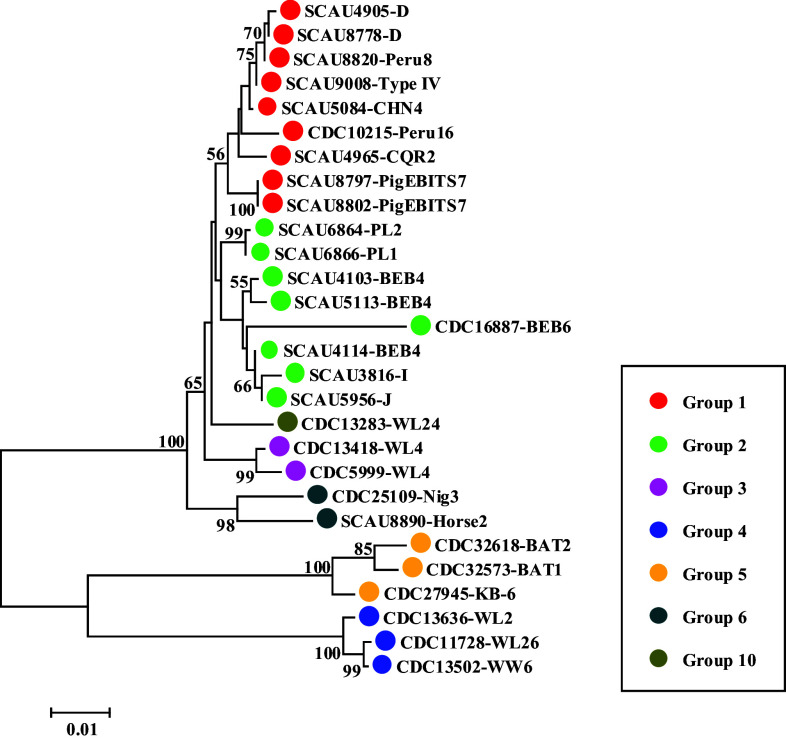
Maximum likelihood tree constructed using concatenated sequences of three genetic loci (ITS, *hp1*, and *tub1*) from *Enterocytozoon bieneusi*. Bootstrap values greater than 50% from 1000 replicates are shown on nodes. Circles of different colors (red, light green, magenta, blue, yellow, dark cyan, and olive) represent the isolates from ITS Groups 1–6 and 10, respectively.

In this study, sequences of 72 isolates of *E. bieneusi* belonging to ITS Groups 1–6 and 10 were obtained from all three genetic loci (ITS, *hp1*, and *tub1*) (Table S3). These sequences were concatenated resulting in an alignment of 1572 bp. The phylogenetic tree based on the concatenated sequences showed that isolates from Group 1–3, 6 and 10 formed a major cluster (Fig. 3). On the other hand, sequences from ITS Groups 4 and 5 were found to be positioned outside of this major cluster in the phylogenetic tree (Fig. 3).

## Discussion

MLST is a conventional genotyping method based on nucleic acid sequence determination. This method directly determines the nucleotide sequence of specific loci of pathogens to analyze their evolution and population genetic characteristics. In this study, we identified two highly polymorphic gene markers *hp1* and *tub1* for high-resolution typing of *E. bieneusi* isolates. Together with the genetic loci previously identified, these two loci became part of MLST tools for genotyping of *E. bieneusi* in various hosts.

The two newly identified markers *hp1* and *tub1* had high sequence polymorphisms and could be used for genetic characterization of *E. bieneusi* isolates from different hosts. Among the 156 *E. bieneusi* ITS positive samples, 83 samples were successfully amplified at the *hp1* locus and produced 30 sequence types. Of the 146 *E. bieneusi* ITS positive samples, 83 samples were successfully amplified at the *tub1* locus and divided into 23 sequence types (Table S1). The sequencing chromatograms of the *E. bieneusi*-positive samples included in the analysis showed no overlapping or mixed peaks. As predicted by our annotation data, there is no intron in these two new markers. All the identified SNPs were located in the coding regions of the two loci. Both the *hp1* and *tub1* genes were single-copy genes in the *E. bieneusi* genome; however, the ploidy level of *E. bieneusi* and whether it undergoes mating and a meiotic cycle are still unclear [30]. Similar to the *ck1* and *swp1* loci, the *hp1* and *tub1* loci had high amplification efficiency and sequence polymorphisms for isolates from ITS Groups 1–6 and 10 [20]. By generating multiple sequence types, it became possible to differentiate isolates within these ITS genotype groups. However, isolates in ITS Group 11 could not be amplified at the *hp1* and *tub1* loci. This supports the previous conclusion of genetic uniqueness of the genotypes in Group 11, which might represent a new *Enterocytozoon* sp. [20]. In addition, genotypes Nig4 and Nig6 from Group 12 that were previously placed in Group 6 could not be amplified at the *hp1* and *tub1* loci, further supporting the notion that these two genotypes are divergent from genotypes in Group 6 [10].

Data generated from this study provide support that there is no obvious genetic differentiation between Groups 1 and 2 genotypes. At the *hp1* locus, most other groups beyond Groups 4 and 5 had genotypes scattered around the *hp1* tree, with both genotypes in Groups 1 and 2 belonging to the largest cluster (Fig. 2a). Similarly, at the *tub1* locus, most genotypes in Group 1 and Group 2 had similar sequences and were placed in the same cluster phylogenetically (Fig. 2b). This result is in agreement with the findings at the *ck1* and *swp1* loci [20]. Previously, it was generally thought that Group 1 genotypes had low host specificity with high zoonotic potential, and Group 2 genotypes had stronger host specificity [17, 24, 29]. However, with the more extensive research on *E. bieneusi* carried out recently, Group 2 genotypes such as BEB4, I, J, and BEB6 were found to experience host range expansion to include humans [11, 18, 22, 25, 33]. The lack of genetic differentiation between Group 1 and Group 2 genotypes at these four loci suggests that genetic recombination might occur among them, consistent with the discoveries in recent research [17, 20]. The two new loci, *tub1* and *hp1*, could serve as complementary markers for studying genetic variation, which can be concatenated with previously reported markers (MS1, MS3, MS4, MS7, *swp1*, and *ck1*) to achieve higher resolution of MLST analysis, providing improved discriminatory power for studying the transmission dynamics, host specificity, and population structure of *E. bieneusi*.

Sequence analyses of the *hp1* and *tub1* loci in this study suggest that genotypes in Group 4 and Group 5 are more divergent from other genotypes within Groups 1–10. At the *hp1* locus, compared to the reference sequence EBI_21704, the Group 4 and Group 5 genotypes had 34–38 SNPs and 28–46 SNPs, respectively. At the *tub1* locus, the genotypes in Group 4 had 40–41 SNPs and the genotypes in Group 5 had 34–36 SNPs compared to the reference sequence EBI_21729. In contrast, other groups had a lower number of SNPs, with 1–28 SNPs at *hp1* and 0–11 SNPs at *tub1* compared to EBI_21704 and EBI_21729, respectively. This observation is supported by phylogenetic sequence analyses from the two loci, where genotypes of Groups 4 and 5 are shown to be distinct from other genotype groups (Fig. 2). Further phylogenetic analysis based on concatenated sequences of all three loci (ITS, *hp1*, and *tub1*) supports this finding (Fig. 3). The ITS genotypes WW6, WL2, and WL26 in Group 4 have been found in raccoons and river otters in China and the USA [6, 14, 27], while genotypes BAT1, BAT2, and KB-6 in Group 5 were identified in straw-colored fruit bats and baboons in Nigeria and Kenya [13, 16]. To further establish the genetic uniqueness of these two groups, it is imperative to conduct future analyses on a larger sample size.

## Conclusion

In conclusion, this study identified two additional genetic loci *hp1* and *tub1,* which can be used to characterize and analyze *E. bieneusi* from different hosts. These loci provide valuable information for understanding the genetic diversity and phylogenetic relationships of *E. bieneusi* isolates. Genotyping based on these loci has confirmed the lack of genetic differentiation between Group 1 and Group 2 genotypes. It has also revealed the genetic uniqueness of genotypes within Groups 4 and 5. These findings highlight the importance of new genetic markers in enabling high-resolution typing of *E. bieneusi* and further studies on the population genetics of *E. bieneusi* and its potential impact on public health in both humans and animals.

## Data Availability

The data supporting the findings of this study are included in the article and its supplementary materials. The nucleotide sequences generated in this study have been deposited in GenBank under accession numbers PP963521 to PP963550 for the *hp1* locus and PP963551 to PP963573 for the *tub1* locus.

## Supplementary material

The supplementary material for this article can be found at https://www.parasite-journal.org/10.1051/parasite/2025002/olm. *Supplementary Table S1*: *Enterocytozoon bieneusi* isolates used in this study and their genetic characteristics at the hypothetical protein 1 (*hp1*) and tubulin 1 (*tub1*) loci. *Supplementary Table S2*: Samples of *Enterocytozoon bieneusi* used in this study and their nested PCR amplification efficiency at the hypothetical protein 1 (*hp1*) and tubulin 1 (*tub1*) loci by internal transcribed spacer (ITS) genotype. *Supplementary Table S3*: *Enterocytozoon bieneusi* isolates that have sequence data from the hypothetical protein 1 (*hp1*) and tubulin 1 (*tub1*) in this study.
