# Supplementary material for: Identification of two new genetic loci for high-resolution genotyping of Enterocytozoon bieneusi
Source: Parasite. 2025 Jan 31;32:6. doi: 10.1051/parasite/2025002 (PMC11784105; doi:10.1051/parasite/2025002)
Supplement: Supplementary file 1 — Supplementary Table S1: Enterocytozoon bieneusi isolates used in this study and their genetic characteristics at the hypothetical protein 1 (hp1) and tubulin 1 (tub1) loci. [file parasite-32-6-s1.pdf]

Table S1

Enterocytozoon bieneusi isolates used in this study and their genetic characteristics at the hypothetical protein 1 (*hp1*) and tubulin 1 (*tub1*) loci

**No.**	**Sample ID**	**Location**	**Animal source**	**ITS Geno-group**	**ITS genotype (GenBank accession no.)**	**hp1 seq.**	**hp1 seq. type (GenBank accession no.)**	**tub1 seq.**	**tub1 seq. type (GenBank accession no.)**
1	SCAU4961	CN (JX)	Bamboo rat	1	CQR2 (KX034075)	EBI_21704 (8 SNPs)	E10 (PP963530)	EBI_21729 (6 SNPs)	E7 (PP963557)
2	SCAU4965	CN (JX)	Bamboo rat	1	CQR2 (KX034075)	EBI_21704 (3 SNPs)	E1 (PP963521)	EBI_21729 (5 SNPs)	E5 (PP963555)
3	SCAU4970	CN (JX)	Bamboo rat	1	CQR2 (KX034075)	neg		EBI_21729 (6 SNPs)	E7 (PP963557)
4	SCAU4988	CN (JX)	Bamboo rat	1	CQR2 (KX034075)	EBI_21704 (8 SNPs)	E10 (PP963530)	EBI_21729 (6 SNPs)	E7 (PP963557)
5	SCAU5001	CN (JX)	Bamboo rat	1	CQR2 (KX034075)	EBI_21704 (8 SNPs)	E10 (PP963530)	EBI_21729 (6 SNPs)	E7 (PP963557)
6	SCAU5013	CN (JX)	Bamboo rat	1	CQR2 (KX034075)	EBI_21704 (8 SNPs)	E10 (PP963530)	neg	
7	SCAU5019	CN (JX)	Bamboo rat	1	CQR2 (KX034075)	EBI_21704 (8 SNPs)	E10 (PP963530)	EBI_21729 (6 SNPs)	E7 (PP963557)
8	SCAU4957	CN (JX)	Bamboo rat	1	CQR2 (KX034075)	neg		EBI_21729 (6 SNPs)	E7 (PP963557)
9	SCAU104	CN (GZ)	Cattle	1	CAF1 (DQ683746)	neg		neg	
10	SCAU5079	CN (SH)	Cattle	1	CHN4 (HM992511)	EBI_21704 (2 SNPs)	E6 (PP963526)	EBI_21729 (3 SNPs)	E2 (PP963552)
11	SCAU5084	CN (SH)	Cattle	1	CHN4 (HM992511)	EBI_21704 (3 SNPs)	E29 (PP963549)	EBI_21729 (4 SNPs)	E23 (PP963573)
12	SCAU6265	CN (CS)	Dog	1	D (KY950534)	neg		neg	
13	SCAU9013	CN (GZ)	Cat	1	D (KY950534)	EBI_21704 (3 SNPs)	E1 (PP963521)	EBI_21729 (4 SNPs)	E1 (PP963551)
14	SCAU4905	CN (GZ)	Cat	1	D (KY950534)	EBI_21704 (4 SNP)	E2 (PP963522)	EBI_21729 (3 SNPs)	E2 (PP963552)
15	SCAU5005	CN (JX)	Bamboo rat	1	D (KY950534)	neg		neg	
16	SCAU4958	CN (JX)	Bamboo rat	1	D (KY950534)	EBI_21704 (3 SNPs)	E1 (PP963521)	EBI_21729 (3 SNPs)	E2 (PP963552)
17	SCAU4959	CN (JX)	Bamboo rat	1	D (KY950534)	EBI_21704 (3 SNPs)	E1 (PP963521)	EBI_21729 (3 SNPs)	E2 (PP963552)
18	SCAU4980	CN (JX)	Bamboo rat	1	D (KY950534)	neg		EBI_21729 (3 SNPs)	E2 (PP963552)
19	SCAU4981	CN (JX)	Bamboo rat	1	D (KY950534)	EBI_21704 (3 SNPs)	E1 (PP963521)	EBI_21729 (3 SNPs)	E2 (PP963552)
20	SCAU5011	CN (JX)	Bamboo rat	1	D (KY950534)	EBI_21704 (3 SNPs)	E1 (PP963521)	EBI_21729 (5 SNPs)	E9 (PP963559)
21	SCAU8778	CN (HN)	Bamboo rat	1	D (KY950534)	EBI_21704 (3 SNPs)	E1 (PP963521)	EBI_21729 (4 SNPs)	E1 (PP963551)

22	SCAU8821	CN (HN)	Bamboo rat	1	D (KY950534)	neg		EBI_21729 (4 SNPs)	E1 (PP963551)
23	SCAU8822	CN (HN)	Bamboo rat	1	D (KY950534)	neg		neg	
24	SCAU8820	CN (HN)	Bamboo rat	1	Peru8 (AY371283)	EBI_21704 (3 SNP)	E1 (PP963521)	EBI_21729 (4 SNPs)	E1 (PP963551)
25	SCAU8797	CN (HN)	Bamboo rat	1	PigEBITS7 (AF348475)	EBI_21704 (7 SNPs)	E11 (PP963531)	EBI_21729 (9 SNPs)	E8 (PP963558)
26	SCAU8796	CN (HN)	Bamboo rat	1	PigEBITS7 (AF348475)	EBI_21704 (7 SNPs)	E11 (PP963531)	neg	
27	SCAU8798	CN (HN)	Bamboo rat	1	PigEBITS7 (AF348475)	neg		neg	
28	SCAU8800	CN (HN)	Bamboo rat	1	PigEBITS7 (AF348475)	neg		neg	
29	SCAU8801	CN (HN)	Bamboo rat	1	PigEBITS7 (AF348475)	EBI_21704 (7 SNPs)	E11 (PP963531)	EBI_21729 (9 SNPs)	E8 (PP963558)
30	SCAU8802	CN (HN)	Bamboo rat	1	PigEBITS7 (AF348475)	EBI_21704 (7 SNPs)	E11 (PP963531)	EBI_21729 (9 SNPs)	E8 (PP963558)
31	SCAU8781	CN (HN)	Bamboo rat	1	Type IV (KX964628)	neg		neg	
32	SCAU153	CN (GZ)	Dog	1	Type IV (KX964628)	EBI_21704 (3 SNPs)	E1 (PP963521)	EBI_21729 (4 SNPs)	E1 (PP963551)
33	SCAU9007	CN (GZ)	Cat	1	Type IV (KX964628)	EBI_21704 (3 SNPs)	E1 (PP963521)	EBI_21729 (3 SNPs)	E2 (PP963552)
34	SCAU8292	CN (GZ)	Cat	1	Type IV (KX964628)	neg		neg	
35	SCAU454	CN (GZ)	Cat	1	Type IV (KX964628)	EBI_21704 (3 SNPs)	E1 (PP963521)	EBI_21729 (4 SNPs)	E1 (PP963551)
36	SCAU4827	CN (GZ)	Cat	1	Type IV (KX964628)	neg		neg	
37	SCAU4843	CN (GZ)	Cat	1	Type IV (KX964628)	EBI_21704 (4 SNPs)	E3 (PP963523)	EBI_21729 (4 SNPs)	E1 (PP963551)
38	SCAU9008	CN (GZ)	Cat	1	Type IV (KX964628)	EBI_21704 (3 SNPs)	E1 (PP963521)	EBI_21729 (3 SNPs)	E2 (PP963552)
39	SCAU9009	CN (GZ)	Cat	1	Type IV (KX964628)	EBI_21704 (3 SNPs)	E1 (PP963521)	EBI_21729 (3 SNPs)	E2 (PP963552)
40	CDC11588	Peru	Guinea pig	1	Peru16 (EF014427)	EBI_21704 (4 SNPs)	E25 (PP963545)	neg	
41	CDC10216	Peru	Guinea pig	1	Peru16 (EF014427)	neg		N/A	
42	CDC11589	Peru	Guinea pig	1	Peru16 (EF014427)	neg		neg	
43	CDC10215	Peru	Human	1	Peru16 (EF014427)	EBI_21704 (4 SNPs)	E25 (PP963545)	EBI_21729 (5 SNPs)	E17 (PP963567)
44	CDC10214	Peru	Human	1	Peru16 (EF014427)	EBI_21704 (4 SNPs)	E25 (PP963545)	EBI_21729 (5 SNPs)	E17 (PP963567)
45	SCAU5107	CN (SH)	Cattle	2	BEB4 (AY331008)	EBI_21704 (2 SNPs)	E6 (PP963526)	EBI_21729 (3 SNPs)	E2 (PP963552)
46	SCAU5108	CN (SH)	Cattle	2	BEB4 (AY331008)	EBI_21704 (2 SNPs)	E6 (PP963526)	EBI_21729 (3 SNPs)	E2 (PP963552)
47	SCAU5111	CN (SH)	Cattle	2	BEB4 (AY331008)	EBI_21704 (2 SNPs)	E6 (PP963526)	EBI_21729 (3 SNPs)	E2 (PP963552)
48	SCAU5113	CN (SH)	Cattle	2	BEB4 (AY331008)	EBI_21704 (2 SNPs)	E6 (PP963526)	EBI_21729 (3 SNPs)	E2 (PP963552)

49	SCAU5200	CN (SH)	Cattle	2	BEB4 (AY331008)	EBI_21704 (2 SNPs)	E6 (PP963526)	EBI_21729 (3 SNPs)	E2 (PP963552)
50	SCAU4084	CN (JS)	Cattle	2	BEB4 (AY331008)	EBI_21704 (8 SNPs)	E7 (PP963527)	EBI_21729 (4 SNPs)	E1 (PP963551)
51	SCAU4103	CN (JS)	Cattle	2	BEB4 (AY331008)	EBI_21704 (5 SNPs)	E8 (PP963528)	EBI_21729 (3 SNPs)	E4 (PP963554)
52	SCAU4114	CN (JS)	Cattle	2	BEB4 (AY331008)	EBI_21704 (8 SNPs)	E7 (PP963527)	EBI_21729 (4 SNPs)	E1 (PP963551)
53	SCAU3816	CN (JS)	Cattle	2	I (AF135836)	EBI_21704 (8 SNPs)	E7 (PP963527)	EBI_21729	E3 (PP963553)
54	SCAU3895	CN (JS)	Cattle	2	I (AF135836)	EBI_21704 (8 SNPs)	E7 (PP963527)	EBI_21729	E3 (PP963553)
55	SCAU3918	CN (JS)	Cattle	2	I (AF135836)	EBI_21704 (8 SNPs)	E7 (PP963527)	EBI_21729	E3 (PP963553)
56	SCAU4017	CN (JS)	Cattle	2	I (AF135836)	EBI_21704 (8 SNPs)	E7 (PP963527)	EBI_21729	E3 (PP963553)
57	SCAU4028	CN (JS)	Cattle	2	I (AF135836)	EBI_21704 (8 SNPs)	E7 (PP963527)	EBI_21729	E3 (PP963553)
58	SCAU4032	CN (JS)	Cattle	2	I (AF135836)	EBI_21704 (8 SNPs)	E7 (PP963527)	neg	
59	SCAU5946	CN (SH)	Cattle	2	J (AF135837)	EBI_21704 (8 SNPs)	E7 (PP963527)	EBI_21729	E3 (PP963553)
60	SCAU5954	CN (SH)	Cattle	2	J (AF135837)	EBI_21704 (8 SNPs)	E7 (PP963527)	EBI_21729	E3 (PP963553)
61	SCAU5955	CN (SH)	Cattle	2	J (AF135837)	EBI_21704 (8 SNPs)	E7 (PP963527)	EBI_21729	E3 (PP963553)
62	SCAU5956	CN (SH)	Cattle	2	J (AF135837)	EBI_21704 (8 SNPs)	E7 (PP963527)	EBI_21729 (4 SNPs)	E1 (PP963551)
63	SCAU5959	CN (SH)	Cattle	2	J (AF135837)	EBI_21704 (8 SNPs)	E7 (PP963527)	EBI_21729	E3 (PP963553)
64	SCAU5960	CN (SH)	Cattle	2	J (AF135837)	EBI_21704 (8 SNPs)	E7 (PP963527)	EBI_21729	E3 (PP963553)
65	SCAU5961	CN (SH)	Cattle	2	J (AF135837)	EBI_21704 (8 SNPs)	E7 (PP963527)	EBI_21729	E3 (PP963553)
66	SCAU5962	CN (SH)	Cattle	2	J (AF135837)	EBI_21704 (8 SNPs)	E7 (PP963527)	EBI_21729	E3 (PP963553)
67	SCAU5163	CN (QH)	Yak	2	J (AF135837)	neg		neg	
68	SCAU6866	CN (HN)	Civet	2	PL1 (MT497890)	EBI_21704 (10 SNPs)	E9 (PP963529)	EBI_21729 (5 SNPs)	E6 (PP963556)
69	SCAU6867	CN (HN)	Civet	2	PL1 (MT497890)	EBI_21704 (10 SNPs)	E9 (PP963529)	neg	
70	SCAU6872	CN (HN)	Civet	2	PL1 (MT497890)	neg		EBI_21729 (5 SNPs)	E6 (PP963556)
71	SCAU4813	CN (HN)	Civet	2	PL1 (MT497890)	EBI_21704 (10 SNPs)	E9 (PP963529)	EBI_21729 (5 SNPs)	E6 (PP963556)
72	SCAU4814	CN (HN)	Civet	2	PL1 (MT497890)	EBI_21704 (10 SNPs)	E9 (PP963529)	EBI_21729 (5 SNPs)	E6 (PP963556)
73	SCAU4816	CN (HN)	Civet	2	PL1 (MT497890)	neg		EBI_21729 (5 SNPs)	E6 (PP963556)
74	SCAU4820	CN (HN)	Civet	2	PL1 (MT497890)	neg		neg	
75	SCAU4822	CN (HN)	Civet	2	PL1 (MT497890)	EBI_21704 (10 SNPs)	E9 (PP963529)	EBI_21729 (5 SNPs)	E6 (PP963556)

76	SCAU6826	CN (HN)	Civet	2	PL1 (MT497890)	EBI_21704 (10 SNPs)	E9 (PP963529)	EBI_21729 (5 SNPs)	E6 (PP963556)
77	SCAU6859	CN (HN)	Civet	2	PL2 (MT497891)	neg		EBI_21729 (5 SNPs)	E6 (PP963556)
78	SCAU6863	CN (HN)	Civet	2	PL2 (MT497891)	neg		neg	
79	SCAU6864	CN (HN)	Civet	2	PL2 (MT497891)	EBI_21704 (10 SNPs)	E9 (PP963529)	EBI_21729 (5 SNPs)	E6 (PP963556)
80	SCAU6865	CN (HN)	Civet	2	PL2 (MT497891)	neg		neg	
81	SCAU6871	CN (HN)	Civet	2	PL2 (MT497891)	EBI_21704 (10 SNPs)	E9 (PP963529)	EBI_21729 (5 SNPs)	E6 (PP963556)
82	SCAU4815	CN (HN)	Civet	2	PL2 (MT497891)	neg		neg	
83	SCAU4823	CN (HN)	Civet	2	PL2 (MT497891)	neg		EBI_21729 (5 SNPs)	E6 (PP963556)
84	SCAU6824	CN (HN)	Civet	2	PL2 (MT497891)	EBI_21704 (10 SNPs)	E9 (PP963529)	EBI_21729 (5 SNPs)	E6 (PP963556)
85	SCAU6827	CN (HN)	Civet	2	PL2 (MT497891)	neg		EBI_21729 (5 SNPs)	E6 (PP963556)
86	CDC6197	USA	Cattle	2	I (AF135836)	EBI_21704 (8 SNPs)	E7 (PP963527)	neg	
87	CDC6161	USA	Cattle	2	J (AF135837)	neg		neg	
88	CDC9269	USA	Cattle	2	PtEb XI (DQ885585)	neg		neg	
89	CDC16887	Peru	Goat	2	BEB6 (EU153584)	EBI_21704 (27 SNPs)	E27 (PP963547)	EBI_21729 (11 SNPs)	E14 (PP963564)
90	CDC6202	USA	Cattle	2	I (AF135836)	EBI_21704 (8 SNPs)	E7 (PP963527)	EBI_21729 (4 SNPs)	E1 (PP963551)
91	CDC7247	USA	Cattle	2	BEB4 (AY331008)	neg		N/A	
92	CDC7248	USA	Cattle	2	BEB4 (AY331008)	neg		N/A	
93	CDC7252	USA	Cattle	2	BEB4 (AY331008)	neg		N/A	
94	CDC7254	USA	Cattle	2	BEB4 (AY331008)	neg		N/A	
95	CDC7278	USA	Cattle	2	I (AF135836)	neg		N/A	
96	CDC16888	Peru	Goat	2	BEB6 (EU153584)	neg		neg	
97	CDC5999	USA	Muskrat	3	WL4 (AY237212)	EBI_21704 (28 SNPs)	E13 (PP963533)	EBI_21729 (8 SNPs)	E13 (PP963563)
98	CDC3548	USA	Muskrat	3	WL4 (AY237212)	EBI_21704 (1 SNP)	E18 (PP963538)	neg	
99	CDC12977	USA	Deer mouse	3	WL25 (KF591686)	neg		neg	
100	CDC13418	USA	Raccoon	3	WL4 (AY237212)	EBI_21704 (15 SNPs)	E12 (PP963532)	EBI_21729 (5 SNPs)	E15 (PP963565)
101	CDC13484	USA	Woodchuck	3	WL22 (KF591684)	neg		neg	
102	CDC13502	USA	Raccoon	4	WW6 (JQ863274)	EBI_21704 (34 SNPs)	E22 (PP963542)	EBI_21729 (40 SNPs)	E16 (PP963566)

103	CDC13636	USA	River otter	4	WL2 (AY237210)	EBI_21704 (35 SNPs)	E23 (PP963543)	EBI_21704 (41 SNPs)	E10 (PP963560)
104	CDC11728	USA	Raccoon	4	WL26 (KF591687)	EBI_21704 (38 SNPs)	E24 (PP963544)	EBI_21704 (41 SNPs)	E11 (PP963561)
105	CDC32573	Nigeria	Straw-colored bat	5	BAT1 (MK007973)	EBI_21704 (34 SNPs)	E15 (PP963535)	EBI_21729 (36 SNPs)	E22 (PP963572)
106	CDC32618	Nigeria	Straw-colored bat	5	BAT2 (MK007974)	EBI_21704 (34 SNPs)	E16 (PP963536)	EBI_21729 (35 SNPs)	E19 (PP963569)
107	CDC27920	Kenya	Baboon	5	KB-6 (JF681180)	EBI_21704 (28 SNPs)	E30 (PP963550)	neg	
108	CDC9175	Portugal	Marmoset	5	PtEb XII (DQ885588)	neg		N/A	
109	CDC27922	Kenya	Baboon	5	KB-6 (JF681180)	EBI_21704 (46 SNPs)	E28 (PP963548)	neg	
110	CDC27923	Kenya	Baboon	5	KB-6 (JF681180)	neg		neg	
111	CDC27929	Kenya	Baboon	5	KB-6 (JF681180)	neg		EBI_21729 (34 SNPs)	E21 (PP963571)
112	CDC27935	Kenya	Baboon	5	KB-6 (JF681180)	neg		EBI_21729 (35 SNPs)	E20 (PP963570)
113	CDC27945	Kenya	Baboon	5	KB-6 (JF681180)	EBI_21704 (38 SNPs)	E17 (PP963537)	EBI_21729 (35 SNPs)	E20 (PP963570)
114	SCAU8890	CN (IM)	Horse	6	Horse2 (GQ406054)	EBI_21704 (10 SNPs)	E4 (PP963524)	EBI_21729 (4 SNPs)	E1 (PP963551)
115	SCAU8906	CN (IM)	Horse	6	Horse2 (GQ406054)	neg		neg	
116	SCAU8913	CN (IM)	Horse	6	Horse2 (GQ406054)	neg		neg	
117	SCAU8915	CN (IM)	Horse	6	Horse2 (GQ406054)	EBI_21704 (13 SNPs)	E5 (PP963525)	EBI_21729 (4 SNPs)	E1 (PP963551)
118	CDC32676	Algeria	Camel	6	Macaque1 (JX000572)	EBI_21704 (9 SNPs)	E26 (PP963546)	neg	
119	CDC32678	Algeria	Camel	6	Camel-2 (LC270279)	EBI_21704 (9 SNPs)	E21 (PP963541)	neg	
120	CDC25109	Nigeria	Human	6	Nig3 (JN997479)	EBI_21704 (10 SNPs)	E19 (PP963539)	EBI_21729 (5 SNPs)	E18 (PP963568)
121	CDC13283	USA	Raccoon	10	WL24 (KF591688)	EBI_21704 (9 SNPs)	E20 (PP963540)	EBI_21729 (9 SNPs)	E12 (PP963562)
122	CDC35253	USA	Prairie dogs	10	Row (KP780977)	EBI_21704 (25 SNPs)	E14 (PP963534)	EBI_21729 (5 SNPs)	E15 (PP963565)
123	CDC35254	USA	Prairie dogs	10	Row (KP780977)	EBI_21704 (25 SNPs)	E14 (PP963534)	EBI_21729 (5 SNPs)	E15 (PP963565)
124	CDC35256	USA	Prairie dogs	10	Row (KP780977)	neg		N/A	
125	CDC35287	USA	Prairie dogs	10	Row (KP780977)	EBI_21704 (25 SNPs)	E14 (PP963534)	EBI_21729 (5 SNPs)	E15 (PP963565)
126	SCAU273	CN (GZ)	Dog	11	PtEb IX (DQ885585)	neg		neg	
127	SCAU964	CN (GZ)	Dog	11	PtEb IX (DQ885585)	neg		neg	
128	SCAU1001	CN (GZ)	Dog	11	PtEb IX (DQ885585)	neg		neg	

129	SCAU1237	CN (ST)	Dog	11	PtEb IX (DQ885585)	neg	neg
130	SCAU1269	CN (ST)	Dog	11	PtEb IX (DQ885585)	neg	neg
131	SCAU1274	CN (ST)	Dog	11	PtEb IX (DQ885585)	neg	neg
132	SCAU1291	CN (ST)	Dog	11	PtEb IX (DQ885585)	neg	neg
133	SCAU982	CN (GZ)	Dog	11	PtEb IX (DQ885585)	neg	neg
134	SCAU983	CN (GZ)	Dog	11	PtEb IX (DQ885585)	neg	neg
135	SCAU1851	CN (GZ)	Cat	11	PtEb IX (DQ885585)	neg	neg
136	SCAU977	CN (GZ)	Dog	11	PtEb IX (DQ885585)	neg	neg
137	SCAU978	CN (GZ)	Dog	11	PtEb IX (DQ885585)	neg	neg
138	SCAU990	CN (GZ)	Dog	11	PtEb IX (DQ885585)	neg	neg
139	SCAU1065	CN (GZ)	Dog	11	PtEb IX (DQ885585)	neg	neg
140	SCAU1120	CN (GZ)	Dog	11	PtEb IX (DQ885585)	neg	neg
141	SCAU1130	CN (GZ)	Dog	11	PtEb IX (DQ885585)	neg	neg
142	SCAU976	CN (GZ)	Dog	11	PtEb IX (DQ885585)	neg	neg
143	SCAU6255	CN (CS)	Dog	11	WW8 (KJ668736)	neg	neg
144	SCAU838	CN (FS)	Dog	11	WW8 (KJ668736)	neg	neg
145	SCAU1849	CN (GZ)	Dog	11	WW8 (KJ668736)	neg	neg
146	SCAU6252	CN (CS)	Dog	11	WW8 (KJ668736)	neg	neg
147	SCAU6256	CN (CS)	Dog	11	WW8 (KJ668736)	neg	neg
148	SCAU6257	CN (CS)	Dog	11	WW8 (KJ668736)	neg	neg
149	CDC33070	Nigeria	Human	12	Nig4 (JN997480)	neg	neg
150	CDC33079	Nigeria	Human	12	Nig6 (JX524505)	neg	neg
151	CDC25005	Nigeria	Human	12	Nig4 (JN997480)	neg	N/A
152	CDC33018	Nigeria	Human	12	Nig6 (JX524505)	neg	neg
153	CDC33074	Nigeria	Human	12	Nig6 (JX524505)	neg	neg
154	CDC33081	Nigeria	Human	12	Nig6 (JX524505)	neg	N/A
155	CDC33090	Nigeria	Human	12	Nig4 (JN997480)	neg	neg

156	CDC33094	Nigeria	Human	12	Nig6 (JX524505)	neg	neg

SCAU, samples from South China Agricultural University, China; CDC, samples from the Centers for Disease Control and Prevention, USA; EBI_21704, reference sequence of the *hp1* locus; EBI_21729, reference sequence of the *tub1* locus; En, different sequence types at each locus; N/A, not available; ITS, internal transcribed spacer; seq., sequence; neg, the result of the experiment was negative; CN, China; JX, Jiangxi; GZ, Guangzhou; SH, Shanghai; CS, Chaoshan; HN, Henan; JS, Jiangsu; QH, Qinghai; IM, Inner Mongolia; ST, Shantou; SZ, Shenzhen; FS, Foshan.
