# Supplementary material for: Identification of two new genetic loci for high-resolution genotyping of Enterocytozoon bieneusi
Source: Parasite. 2025 Jan 31;32:6. doi: 10.1051/parasite/2025002 (PMC11784105; doi:10.1051/parasite/2025002)
Supplement: Supplementary file 2 — Supplementary Table S2: Samples of Enterocytozoon bieneusi used in this study and their nested PCR amplification efficiency at the hypothetical protein 1 (hp1) and tubulin 1 (tub1) loci by internal transcribed spacer (ITS) genotype. [file parasite-32-6-s2.pdf]

Table S2

Samples of *Enterocytozoon bieneusi* used in this study and their nested PCR amplification efficiency at the hypothetical protein 1 (*hp1*) and tubulin 1 (*tub1*) loci by internal transcribed spacer (ITS) genotype.

**Group**	**ITS genotype**	**Host (number of samples)**	**Source location**	**No. of positives**	
				hp1	*tub1*
**1**	CAF1	Cattle (1)	CN (GZ)	0/1	0/1
	CHN4	Cattle (2)	CN (SH)	2/2	2/2
	CQR2	Bamboo rat (8)	CN (JX)	6/8	7/8
	D	Bamboo rat (9), dog (1), cat (2)	CN (GZ), CN (JX), CN (CS), CN (HN)	7/12	9/12
	Peru8	Bamboo rat (1)	CN (HN)	1/1	1/1
	Peru16	Human (2), Guinea pig (3)	Peru	3/5	2/4
	PigEBITS7	Bamboo rat (6)	CN (HN)	4/6	3/6
	Type IV	Bamboo rat (1), dog (1), cat (8)	CN (GZ)	6/9	6/9
	Subtotal	Cattle (3), human (2), guinea pig (3), bamboo rat (25), dog (2), cat (10)	CN (GZ), CN (SH), CN (JX), CN (CS), CN (HN), Peru	29/44	30/43
**2**	BEB4	Cattle (12)	CN (SH), USA	8/12	8/8
	BEB6	Goat (2)	Peru	1/2	1/2
	I	Cattle (9)	CN (JS), USA	8/9	6/8
	J	Cattle (10)	CN (SH), USA	8/10	8/10
	PtEb XI	Cattle (1)	Portugal	0/1	0/1
	PL1	Civet (9)	CN (HN)	6/9	7/9
	PL2	Civet (9)	CN (HN)	3/9	6/9

	Subtotal	Cattle (32), goat (2), civet (18)	CN (SH), USA, CN (JS), Peru, Portugal, CN (HN)	34/52	36/47
**3**	WL4	Muskrat (2), Raccoon (1)	USA	3/3	2/3
	WL22	Woodchuck (1)	USA	0/1	0/1
	WL25	Deer mouse (1)	USA	0/1	0/1
	Subtotal	Muskrat (2), raccoon (1), woodchuck (1), deer mouse (1)	USA	3/5	2/5
**4**	WL2	River otter (1)	USA	1/1	1/1
	WL26	Raccoon (1)	USA	1/1	1/1
	WW6	Raccoon (1)	USA	1/1	1/1
	Subtotal	River otter (1), raccoon (2)	USA	3/3	3/3
**5**	BAT1	Straw-colored bat (1)	Nigeria	1/1	1/1
	BAT2	Straw-colored bat (1)	Nigeria	1/1	1/1
	KB-6	Baboon (6)	Kenya	3/6	3/6
	PtEb XII	Marmoset (1)	Portugal	0/1	-
	Subtotal	Baboon (6), straw-colored bat (2), marmoset (1)	Kenya, Nigeria, Portugal	5/9	5/8
**6**	Horse2	Horse (4)	CN (IM)	2/4	2/4
	Nig3	Human (1)	Nigeria	1/1	1/1
	Macaque1	Camel (1)	Algeria	1/1	0/1
	Camel-2b	Camel (1)	Algeria	1/1	0/1
	Subtotal	Human (1), camel (2), horse (4)	Nigeria, Algeria, CN (IM)	5/7	3/7
**10**	Row	Prairie dogs (4)	USA	3/4	3/3
	WL24	Raccoon (1)	USA	1/1	1/1
	Subtotal	Raccoon (1), prairie dogs (4)	USA	4/5	4/4

**11**	PtEb IX	Dog (26), Cat (1)	CN (GZ), CN (CS)	0/17	0/17
	WW8	Dog (26)	CN (GZ), CN (CS)	0/6	0/6
	Subtotal	Dog (52), cat (1)	CN (GZ), CN (CS)	0/23	0/23
**12**	Nig4	Human (3)	Nigeria	0/3	0/2
	Nig6	Human (5)	Nigeria	0/5	0/4
	Subtotal	Human (8)	Nigeria	0/8	0/6
**Total**				83/156	83/146

CN, China; JX, Jiangxi; GZ, Guangzhou; SH, Shanghai; CS, Chaoshan; HN, Henan; JS, Jiangsu; QH, Qinghai; IM, Inner Mongolia; ST, Shantou; SZ, Shenzhen; FS, Foshan.
