# Supplementary material for: Identification of two new genetic loci for high-resolution genotyping of Enterocytozoon bieneusi
Source: Parasite. 2025 Jan 31;32:6. doi: 10.1051/parasite/2025002 (PMC11784105; doi:10.1051/parasite/2025002)
Supplement: Supplementary file 3 — Supplementary Table S3: Enterocytozoon bieneusi isolates that have sequence data from the hypothetical protein 1 (hp1) and tubulin 1 (tub1) in this study. [file parasite-32-6-s3.pdf]

Table S3

Enterocytozoon bieneusi isolates that have sequence data from the hypothetical protein 1 (*hp1*) and tubulin 1 (*tub1*) in this study.

No.	Sample ID	Location	Animal source	Genogroup	ITS	*hp1*	*tub1*
1	SCAU4961	CN (JX)	Bamboo rat	1	CQR2	E10	E7
2	SCAU4965	CN (JX)	Bamboo rat	1	CQR2	E1	E5
3	SCAU4988	CN (JX)	Bamboo rat	1	CQR2	E10	E7
4	SCAU5001	CN (JX)	Bamboo rat	1	CQR2	E10	E7
5	SCAU5019	CN (JX)	Bamboo rat	1	CQR2	E10	E7
6	SCAU5079	CN (SH)	Cattle	1	CHN4	E6	E2
7	SCAU5084	CN (SH)	Cattle	1	CHN4	E29	E23
8	SCAU9013	CN (GZ)	Cat	1	D	E1	E1
9	SCAU4905	CN (GZ)	Cat	1	D	E2	E2
10	SCAU4958	CN (JX)	Bamboo rat	1	D	E1	E2
11	SCAU4959	CN (JX)	Bamboo rat	1	D	E1	E2
12	SCAU4981	CN (JX)	Bamboo rat	1	D	E1	E2
13	SCAU5011	CN (JX)	Bamboo rat	1	D	E1	E9
14	SCAU8778	CN (HN)	Bamboo rat	1	D	E1	E1
15	SCAU8820	CN (HN)	Bamboo rat	1	Peru8	E1	E1
16	SCAU8797	CN (HN)	Bamboo rat	1	PigEBITS7	E11	E8
17	SCAU8801	CN (HN)	Bamboo rat	1	PigEBITS7	E11	E8
18	SCAU8802	CN (HN)	Bamboo rat	1	PigEBITS7	E11	E8
19	SCAU153	CN (GZ)	Dog	1	Type IV	E1	E1
20	SCAU9007	CN (GZ)	Cat	1	Type IV	E1	E2
21	SCAU454	CN (GZ)	Cat	1	Type IV	E1	E1
22	SCAU4843	CN (GZ)	Cat	1	Type IV	E3	E1
23	SCAU9008	CN (GZ)	Cat	1	Type IV	E1	E2
24	SCAU9009	CN (GZ)	Cat	1	Type IV	E1	E2
25	CDC10215	Peru	Human	1	Peru16	E25	E17

26	CDC10214	Peru	Human	1	Peru16	E25	E17
27	SCAU5107	CN (SH)	Cattle	2	BEB4	E6	E2
28	SCAU5108	CN (SH)	Cattle	2	BEB4	E6	E2
29	SCAU5111	CN (SH)	Cattle	2	BEB4	E6	E2
30	SCAU5113	CN (SH)	Cattle	2	BEB4	E6	E2
31	SCAU5200	CN (SH)	Cattle	2	BEB4	E6	E2
32	SCAU4084	CN (JS)	Cattle	2	BEB4	E7	E1
33	SCAU4103	CN (JS)	Cattle	2	BEB4	E8	E4
34	SCAU4114	CN (JS)	Cattle	2	BEB4	E7	E1
35	SCAU3816	CN (JS)	Cattle	2	I	E7	E3
36	SCAU3895	CN (JS)	Cattle	2	I	E7	E3
37	SCAU3918	CN (JS)	Cattle	2	I	E7	E3
38	SCAU4017	CN (JS)	Cattle	2	I	E7	E3
39	SCAU4028	CN (JS)	Cattle	2	I	E7	E3
40	SCAU5946	CN (SH)	Cattle	2	J	E7	E3
41	SCAU5954	CN (SH)	Cattle	2	J	E7	E3
42	SCAU5955	CN (SH)	Cattle	2	J	E7	E3
43	SCAU5956	CN (SH)	Cattle	2	J	E7	E1
44	SCAU5959	CN (SH)	Cattle	2	J	E7	E3
45	SCAU5960	CN (SH)	Cattle	2	J	E7	E3
46	SCAU5961	CN (SH)	Cattle	2	J	E7	E3
47	SCAU5962	CN (SH)	Cattle	2	J	E7	E3
48	SCAU6866	CN (HN)	Civet	2	PL1	E9	E6
49	SCAU4813	CN (HN)	Civet	2	PL1	E9	E6
50	SCAU4814	CN (HN)	Civet	2	PL1	E9	E6
51	SCAU4822	CN (HN)	Civet	2	PL1	E9	E6
52	SCAU6826	CN (HN)	Civet	2	PL1	E9	E6
53	SCAU6864	CN (HN)	Civet	2	PL2	E9	E6
54	SCAU6871	CN (HN)	Civet	2	PL2	E9	E6

55	SCAU6824	CN (HN)	Civet	2	PL2	E9	E6
56	CDC16887	Peru	Goat	2	BEB6	E27	E14
57	CDC6202	USA	Cattle	2	I	E7	E1
58	CDC5999	USA	Muskrat	3	WL4	E13	E13
59	CDC13418	USA	Raccoon	3	WL4	E12	E15
60	CDC13502	USA	Raccoon	4	WW6	E22	E16
61	CDC13636	USA	River otter	4	WL2	E23	E10
62	CDC11728	USA	Raccoon	4	WL26	E24	E11
63	CDC32573	Nigeria	Straw-colored bat	5	BAT1	E15	E22
64	CDC32618	Nigeria	Straw-colored bat	5	BAT2	E16	E19
65	CDC27945	Kenya	Baboon	5	KB-6	E17	E20
66	SCAU8890	CN (IM)	Horse	6	Horse2	E4	E1
67	SCAU8915	CN (IM)	Horse	6	Horse2	E5	E1
68	CDC25109	Nigeria	Human	6	Nig3	E19	E18
69	CDC13283	USA	Raccoon	10	WL24	E20	E12
70	CDC35253	USA	Prairie dogs	10	Row	E14	E15
71	CDC35254	USA	Prairie dogs	10	Row	E14	E15
72	CDC35287	USA	Prairie dogs	10	Row	E14	E15

SCAU, samples from South China Agricultural University, China; CDC, samples from the Centers for Disease Control and Prevention, USA; En, different sequence types at each locus; ITS, internal transcribed spacer; CN, China; JX, Jiangxi; GZ, Guangzhou; SH, Shanghai; CS, Chaoshan; HN, Henan; JS, Jiangsu; QH, Qinghai; IM, Inner Mongolia; ST, Shantou; SZ, Shenzhen; FS, Foshan.
